# EXACT: EXercise or Advice after ankle fraCTure. Design of a randomised controlled trial

**DOI:** 10.1186/1471-2474-12-148

**Published:** 2011-07-05

**Authors:** Paula R Beckenkamp, C Christine Lin, Robert D Herbert, Marion Haas, Kriti Khera, Anne M Moseley

**Affiliations:** 1 The George Institute for Global Health, Sydney Medical School, The University of Sydney, PO Box M201, Missenden Road Sydney, New South Wales 2000, Australia; 2 Centre for Health Economics Research and Evaluation (CHERE), University of Technology, Sydney, PO Box 123, Broadway, New South Wales 2007, Australia

## Abstract

**Background:**

Ankle fractures are common. Management of ankle fractures generally involves a period of immobilisation followed by rehabilitation to reduce pain, stiffness, weakness and swelling. The effects of a rehabilitation program are still unclear. However, it has been shown that important components of rehabilitation programs may not confer additional benefits over exercise alone. The primary aim of this trial is to determine the effectiveness and cost-effectiveness of an exercise-based rehabilitation program after ankle fracture, compared to advice alone.

**Methods/Design:**

A pragmatic randomised trial will be conducted. Participants will be 342 adults with stiff, painful ankles after ankle fracture treated with immobilisation. They will be randomly allocated using a concealed randomisation procedure to either an *Advice *or *Rehabilitation *group. Participants in the *Advice *group will receive verbal and written advice about exercise at the time of removal of immobilisation. Participants in the *Rehabilitation *group will be provided with a 4-week rehabilitation program that is designed, monitored and progressed by a physiotherapist, in addition to verbal and written advice. Outcomes will be measured by a blinded assessor at 1, 3 and 6 months. The primary outcomes will be activity limitation and quality-adjusted life years.

**Discussion:**

This pragmatic trial will determine if a rehabilitation program reduces activity limitation and improves quality of life, compared to advice alone, after immobilisation for ankle fracture.

## Background

Ankle fracture, fracture of the distal tibia and/or fibula, is the second most common ankle injury after ankle sprain [1]. The incidence of ankle fracture is at least 5 per 10,000 person-years across all ages [2,3], but is higher in young men (13 to 28 per 10,000 person-years) and older women (16 to 20 per 10,000 person-years). The orthopaedic management depends on the severity of the fracture, but generally involves immobilisation of the ankle in a cast or a brace for about 6 weeks [4]. Surgical fixation is required in about 50% of cases [5,6].

The disabling sequelae of ankle fracture and the subsequent immobilisation frequently affect people's ability to return to work and sport. Our recent study showed that people with ankle fracture had significant activity limitation after the period of immobilisation (mean Lower Extremity Functional Scale score of 33.6, where no activity limitation receives a score of 80) and one-third were unable to walk without crutches [5]. Impairments include pain [5,6], stiffness [7,8], weakness [9-11] and swelling [12,13]. Rehabilitation programs are often provided to address these sequelae and can commence as soon as the fracture has been stabilised (i.e. during the period of immobilisation) or, more often, after the period of immobilisation (i.e. when bone union has occurred). Typically a post-immobilisation program consists of stretch, manual therapy, exercise, gait training and advice [5,6].

There is limited evidence on the effects of rehabilitation interventions for ankle fracture. A Cochrane systematic review has evaluated the effects of rehabilitation interventions applied during or after the period of immobilisation. It concluded that commencing weight bearing during the immobilisation period and wearing a brace that can be removed to allow gentle ankle exercise may improve outcome after ankle fracture [14].

The Cochrane review identified only two randomised controlled trials which investigated effects of rehabilitation after the immobilisation period [6,15]. Two other trials have been recently completed [5,16]. These trials concluded that adding passive stretch or manual therapy to an exercise program does not improve clinical or economic outcomes compared to exercise alone [5,6,15], and found no overall benefit of participating in an exercise program [16]. However, in the trial that compared an exercise program to usual care, benefits of the exercise program could have been underestimated as there was a high uptake of physiotherapy (a mean of 7 physiotherapy appointments) by participants from the usual care group [16]. In addition, the trial only included people whose ankle had been treated surgically [16].

It is still unclear if some sub-groups could benefit more from rehabilitation than others. Nilsson et al [16] compared the effects of rehabilitation on Olerud-Molander Ankle Score (range 0 to 100) in those aged < 40 years and those ≥ 40. There was no difference in effects of rehabilitation at 6 months. However the effect of rehabilitation at 12 months was greater in people aged < 40 than in those aged ≥ 40 (mean difference in effect 16.9 points, 95% CI 5.6 to 28.2). This finding should be considered provisional until it is replicated in other studies. We postulate that participation in a rehabilitation program may particularly benefit older women (aged over 50 years) as they are more likely to suffer from osteoporosis [17], and physical performance [18] and physical activity [19] also decline with age. The severity of fracture has also been found to influence outcome in ankle fracture [20], which raises the idea that rehabilitation should be offered selectively. In this case, it could be argued that people with more severe fracture would have worse outcomes and, consequently, would benefit more from a rehabilitation program.

The prediction of outcome after ankle fracture is relatively imprecise. We have previously shown that out of eight variables, pain and dorsiflexion range of motion soon after the period of immobilisation were the only independent predictors of outcome, but explained only 9 to 12% of the variance in activity limitation in the short- to medium-term [21]. Other variables (e.g. psychological factors) may be better predictors of outcome after ankle fracture. It has been shown that psychological variables (including pain catastrophising and depression) appear to be important predictors of outcome in people with other musculoskeletal conditions [22]. This issue has not been examined after ankle fracture.

The primary aims of the trial to be conducted are, therefore, to determine (a) the effectiveness and cost-effectiveness of rehabilitation, compared to simple advice and (b) if effects of rehabilitation are influenced by fracture severity or by the age and gender of participants. A secondary aim is to identify predictors of outcome after ankle fracture.

## Methods/Design

**Figure 1:**
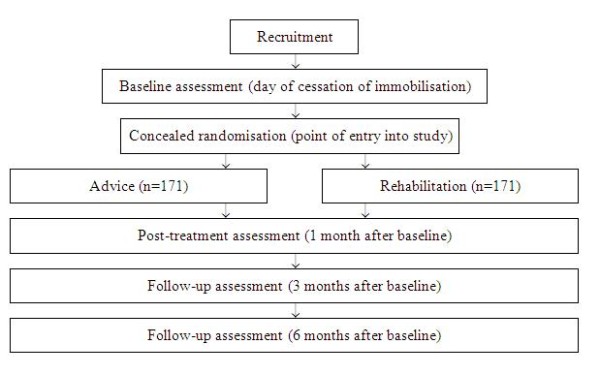
**Experimental protocol**.

The trial will be an assessor-blinded pragmatic randomised controlled trial. Three hundred and forty two participants will be randomly allocated to one of two groups: an *Advice *group and a *Rehabilitation *group (~171 in each group). Outcomes will be assessed at baseline, 1, 3 and 6 months by a blinded assessor (Figure 1).

### Participants

Participants will be recruited from four hospitals. The inclusion criteria will be:

• ankle fracture treated with immobilisation (e.g. cast, brace), with or without surgical fixation

• immobilisation removed on the day of recruitment

• approval received from orthopaedic specialist to weight-bear as tolerated or partial weight-bear

• reduced ankle dorsiflexion range of motion (at least 30 mm less motion compared to the non-fractured leg using the weight-bearing lunge method) [23]

• at least 2 out of 10 pain in the ankle when up to 50% of body weight is borne through the affected leg

• completed skeletal growth (i.e. no evidence of epiphyseal cartilage in the tibia in x-rays taken for fracture management)

• no concurrent pathologies (e.g. symptomatic osteoarthritis, stroke, other fractures) which affect the ability to perform everyday tasks or the measurement procedures used in this trial, and

• informed consent obtained.

### Recruitment sites

Participants will be recruited from the fracture clinics of four public hospitals in Sydney, Australia: Royal North Shore Hospital, Prince of Wales Hospital, Royal Prince Alfred Hospital and Ryde Hospital. Royal North Shore Hospital, Prince of Wales Hospital and Royal Prince Alfred Hospital are major teaching hospitals with more than 500 beds. Ryde Hospital is a smaller teaching hospital of close to 200 beds. At these hospitals the fracture clinics are staffed by physiotherapists, who are responsible for the application and removal of immobilisation and the provision of advice about rehabilitation after orthopaedic clearance.

### Randomisation

Randomisation will be conducted using a telephone-based randomisation service provided by the Australian National Health and Medical Research Council (NHMRC) Clinical Trials Centre. This ensures concealment of the allocation schedule. Allocation will be stratified by site and blocked within strata using random permuted blocks.

### Interventions

Participants in the *Advice *group will be given advice in a single session in the fracture clinic, after removal of immobilisation and after consultation with the treating orthopaedic specialist. A physiotherapist will advise the participant to do exercises that involve ankle movement in non-weight-bearing positions and will explain how to perform these exercises and how to progressively reduce the use of walking aids. The participant will be given a handout that summarises this advice with text and figures.

Participants in the *Rehabilitation *group will receive the same advice but will also participate in an exercise program that is designed, monitored and progressed by a physiotherapist, with participants encouraged to perform a carefully structured exercise program at home. Three types of exercises will be prescribed: ankle mobility and strengthening exercises, stepping exercises, and exercises involving weight-bearing and balancing on the affected leg. These exercises are routinely prescribed after immobilisation for ankle fracture and were used in our recently completed trials [5,6]. Exercise cards have been developed to standardise the exercises used. Participants will also receive gait training and ongoing advice about returning to usual work and leisure activities. In keeping with the pragmatic orientation of the trial, participating physiotherapists will not be prevented from administering stretches or manual therapy. The rehabilitation program will be provided during two sessions in week one and in one session from weeks two to four; further consultations will be at the discretion of the physiotherapist. Participants will be discharged by their physiotherapist when they achieve their pre-fracture function, reach a plateau in their progress, or choose to discontinue the treatment.

When appropriate, participants in both groups will be instructed to use ice for pain relief and compression and elevation for management of swelling. A compression bandage will be provided if required. Treatments will be administered by registered physiotherapists who will be trained to provide the *Advice *and *Rehabilitation *interventions in accordance with the trial protocol.

### Outcome assessment

Outcomes will be assessed at baseline and 1, 3 and 6 months later. Outcome data will be obtained from all randomised participants, in so far as this is possible, regardless of compliance with the trial protocol. Outcome measurements will be made by an assessor blinded to allocation. The participants, as well as physiotherapists and investigators involved in the trial, will be asked to avoid giving information that could reveal participant's allocation to the blinded assessor. After each assessment the assessor will be asked if he or she was blinded to allocation and will be asked to guess which group the participant was allocated to.

At the baseline assessment, demographic and injury details will be recorded. Classification of fracture severity (severe or not severe) will be based on the number of malleoli fractured [20] and the presence of dislocation: bi- or tri-malleolar fractures and any fractures with dislocation will be classified as severe. The assessor will classify fracture severity by examining the radiology entries in the hospital records or, if these are not available, by examining plain radiographs taken before and after the fracture reduction. Two psychological scales will also be used: depression, anxiety and stress will be measured using the Depression, Anxiety, Stress Scales 21-item [24], and pain catastrophising will be assessed using the Pain Catastrophising Scale [25].

The primary outcomes will be activity limitation and quality-adjusted life years. Activity limitation will be measured with the Lower Extremity Functional Scale [26] which involves the participant rating the degree of difficulty in performing 20 functional activities on a 5-point scale ranging from 0 ('extreme difficulty or unable to perform activity') to 4 ('no difficulty'). The scale has excellent test-retest reliability (intraclass correlation coefficient (ICC) 0.94), is sensitive to change and has high internal consistency and construct and concurrent validity for people with ankle fracture [27]. Quality-adjusted life years will be measured by the Assessment of Quality of Life instrument, which is designed to measure health-related quality of life and to be the descriptive system for a multi-attribute utility instrument. The Assessment of Quality of Life measures five dimensions: illness, independent living, social relationships, physical senses and psychological well-being, all of which have been shown to be orthogonal and unidimensional [28]. The Assessment of Quality of Life has been shown to be internally consistent (alpha = 0.81) and has a comparative fit index of 0.90 [28], and is reliable and more sensitive to health status than other multi-attribute utility instruments [29].

Secondary outcomes will be the number of days to pain-free walking, the number of days to return to full pre-fracture work, return to pre-fracture work and leisure activities, ankle dorsiflexion range of motion, pain, walking speed, physical activity, and global perceived effect of treatment (Table 1).

**Table 1:** Secondary outcome measures assessed at baseline, 1, 3 and 6 months, unless otherwise stated.

Outcome	Description of assessment
Number of days to pain-free walking	Participants will be given a calendar to mark the first day they can walk pain-free for 10 meters to calculate the number of days elapsed from the day of randomisation to pain-free walking [5].
Number of days to return to full pre- fracture work	Participants who worked prior to fracture will be given a calendar to mark the first day they return to their pre-fracture work to calculate the number of days elapsed from the day of randomisation to return to full pre-fracture work.
Return to pre-fracture work and leisure	Self-reported percentage return to full pre-fracture work and leisure, where 0% is 'not participating at all' and 100% is 'returned to full level'.
Ankle dorsiflexion range of motion	Measured using the weight-bearing lunge method [23] at baseline and 1-month.
Pain	Pain on equal weight-bearing and on stair descent measured using a numerical rating scale (0 to 10), where 0 is 'no pain' and 10 is 'worst pain you ever had', at baseline and 1-month.
Walking speed	Unaided walking speed over a 10 m distance using a stop watch at baseline and 1-month.
Physical activity	Physical activity will be measured using the International Physical Activity Questionnaire-Short Form [39]. This questionnaire will be used to classify participants into one of the three activity levels (low, moderate or high) and calculate the metabolic equivalent (MET) minutes per week.
Global perceived	Perceived effect of treatment will be measured on an 11-point scale from -5,
effect of treatment	'vastly worse', to +5, 'completely recovered' at 1, 3 and 6 months.

Participants in the *Rehabilitation *and *Advice *groups will complete exercise diaries. Physiotherapists treating participants in the *Rehabilitation *group will complete treatment logs. Participants in the *Advice *group will be asked at each assessment if they sought rehabilitation or physiotherapy services. Participants' perceptions of the credibility of the interventions will be determined by questions administered to all participants at the 6-month follow-up. At the 6-month assessment, participants will also be asked open-ended questions about adverse events.

### Economic evaluation

The economic evaluation will consist of cost-effectiveness and cost-utility analyses. Costs will be measured in terms of direct costs to the health system and out-of-pocket costs to the participants over a 6-month period, and collected in a questionnaire at the 1, 3 and 6 month follow-up period. From an economic perspective, costs are measured by resource use. This allows the identification of the opportunity cost of resources used, that is, what could have been achieved had those resources been allocated to the best alternative use. In this trial the costs of the interventions and any out-of-pocket costs incurred by participants will be identified, measured and valued. Table 2 indicates the type of resources which will be captured, the sources of data, and proposed methods of valuation.

**Table 2:** Assessment of resource use.

Type of resource	Method of assessment	Method of valuation
Physiotherapists' time	Physiotherapists' report	Salary rates plus on-costs for physiotherapists using published prices
Equipment	Questionnaires at 1, 3, 6 months	Manufacturer's price (depreciated over 3 years)
Medication, visits to general practitioners and other health professionals, hospitalisation, visits to emergency department	Questionnaires at 1, 3, 6 months	Published prices (e.g. Pharmaceutical Benefits Scheme and Medicare Benefits Schedule reimbursement) and/or actual costs to participants
Visits to community services or alternative or complementary health practitioners	Questionnaires at 1, 3, 6 months	Actual costs to participants

The cost-effectiveness analysis will use the Lower Extremity Functional Scale [26] as a measure of effectiveness. The cost-utility analysis will use the Assessment of Quality of Life instrument as a measure of utility.

### Statistical analysis

Analyses will be conducted by 'intention-to-treat' (i.e. all available data from all randomised participants will be analysed in the group to which the participant was allocated). Multiple imputation will be used for missing data. To test the effects of intervention on continuous outcomes (activity limitation, quality of life, return to pre-fracture work and leisure, physical activity (metabolic equivalent minutes per week), ankle range of motion, pain, walking speed, and global perceived effect of treatment), between-group comparisons will be conducted using longitudinal mixed models [30,31]. The independent variables will be a dummy-coded variable indicating group membership, the time at which the measurement was taken (four times, dummy-coded as three variables, with the baseline time as the referent category), and the three time-by-group interactions. The effect of rehabilitation at each of the three follow-up time points is estimated with the relevant interaction term. The model will incorporate random intercepts to account for the dependence of repeated measures. Survival analysis will be used to estimate between-group differences in days to pain-free walking and days to return to full pre-fracture work. Odds ratios will be calculated for physical activity (level), satisfaction with trial treatment, and adverse events.

In a second analysis, designed to test the influence of fracture severity on treatment response, additional terms (fracture severity and the interactions of fracture severity with the group and time variables) will be entered into the model. The effect of fracture severity on treatment response will be determined by examining the interactions between group membership, fracture severity and the time variables. A similar analysis will test the influence of participant age and gender on treatment response. Participants will be divided into women aged over 50 and others. Again, additional terms (age/gender and the interactions of age/gender with the group and time variables) will be entered into the model. The effect of age/gender on treatment response will be determined by examining the interactions between group membership, age/gender and the time variables.

The primary conclusions about effectiveness of rehabilitation will be based on between-group comparisons of activity limitation and quality of life at 3 months. The primary conclusions about whether fracture severity and age and gender influence the effectiveness of intervention will be based on the interactions between these factors and effects of rehabilitation for activity limitation and quality of life at 3 months.

The economic evaluation will examine differences between participants in the *Rehabilitation *and *Advice *groups in terms of costs incurred and changes in perceived activity limitation (cost-effectiveness analysis) or utility gained (cost-utility analysis). The incremental cost-effectiveness (utility) ratio (ICER) will be calculated as: ICER = (*C_R _*- *C_A_*)/(*U_R _*- *U_A_*), where C is average cost, U is the average effectiveness or utility score, and subscripts R and A denote the *Rehabilitation *and *Advice *arms. The *Rehabilitation *program can be said to be cost-effective relative to *Advice *about exercise if it (a) produces less activity limitation or greater utility at a lower cost or (b) the cost per activity limitation avoided or per quality-adjusted life years gained (i.e. the incremental cost-effectiveness (utility) ratio) is less than some threshold value (e.g. $50,000). Bias-corrected bootstrapped estimates (1,000 replications) will be used to test for the difference in mean costs and to obtain 95% confidence intervals for between-group differences in mean costs. Between-group differences in utilisation will be tested using Fisher's exact test. Sensitivity analyses will be undertaken to explore the robustness and validity of the results. Both costs and outcomes will be varied in line with results from similar studies reported in the literature and the upper and lower limits of estimates from this trial.

To establish the predictors of outcome after ankle fracture, univariate linear regression will be used to examine the relationship between seven baseline variables (fracture severity, pain, ankle range of motion, mobility, depression, anxiety and stress, and pain catastrophising) and activity limitation at 1-month and 6 months after removal of immobilisation. A multivariate linear prediction model will be developed using methods described by Harrell [32].

### Sample size

A sample of 76 participants (38 per group) would provide an 80% probability of detecting a difference between the group means of 10 points on the 80-point Lower Extremity Functional Scale (assuming a SD of 15 points, based on data from our recently completed trials [5,6]). This sample size will also provide 80% probability of detecting a difference between the group means of 2.75 points on the 45-point Assessment of Quality of Life scale (assuming a SD of 4 points, based on data from our recently completed trial [5]). Effects smaller than these are unlikely to be considered clinically worthwhile. In our calculations we assumed an alpha of 0.05, and we allowed for 5% loss to follow-up. We conservatively ignored the extra precision conferred by the longitudinal design.

In order to power the trial for analyses of the interactions with fracture severity and age/gender of participants we need to inflate the sample size by a factor of 2 + *k *+ 1/*k*, where *k *is the sub-group ratio. We anticipate the sub-group ratio for both *severe*: *less severe *fracture and *women aged over 50*: *others *is ~1:2 (based on our recently completed studies [5,6]). Thus, a sample of 72 × 4.5 = 342 participants (171 per group) will be recruited.

### Ethics

The trial will be conducted in Sydney, Australia following the principles of the Helsinki declaration and in accordance with the Australia's National Statement on Ethical Conduct in Human Research. Participants will receive information about the study protocol before providing their written consent to be in the study. All data collected will be confidential. This study has ethical approval from the Hawkesbury Human Ethics Committee of Northern Sydney Central Coast Health, Sydney, Australia, and site specific approvals from the ethics committee of each participating site (Prince of Wales Hospital, Ryde Hospital, Royal North Shore Hospital and Royal Prince Alfred Hospital). This trial is registered at the Australian New Zealand Clinical Trials Registry ACTRN12610000979055.

## Discussion

The EXACT trial will provide evidence on the effectiveness of rehabilitation after ankle fracture. Participants receiving the *Rehabilitation *intervention will receive an individually tailored and exercise-based physiotherapy program and participants receiving the *Advice *intervention will not, because the intent is to compare the effects of these two alternatives when they are administered as usually applied in the clinic. We will not match the number of treatment sessions for the *Advice *group because, given the pragmatic orientation of the trial, it is more important to replicate current clinical practice. A pragmatic trial design [33,34] was chosen to enable easy translation of the research findings into clinical practice.

Our trial incorporates features that minimise bias, including concealed random allocation and blinded data analysis [35]. Blinding of outcome assessment may be particularly important in exercise trials such as ours, where the blinding of participants and therapists is not possible [36]. The primary outcomes (activity limitation, quality-adjusted life years etc) will be self-reported by participants who are not blinded. However the assessors who elicit primary outcome data, and who collect secondary outcome data such as ankle dorsiflexion range of motion and walking speed, will be unaware of group allocation. Consequently the assessment of outcome is partially blinded. Procedures recently suggested to achieve blinding in pragmatic physiotherapy trials will be used in our trial, including asking participants not to reveal their group allocation to assessors when booking follow-up appointments and at the start of each assessment, and restricting assessor access to trial data which indicates group allocation (computer files will be password protected and paperwork will be in a separate locked filing cabinet) [37]. We will also confirm the success of assessor blinding by asking the assessor if he or she was blinded to allocation and to guess which group the participant was allocated to after each assessment.

The sample size will be large enough to provide precise estimates of treatment effects. Importantly, the trial is adequately powered to investigate whether the effects of rehabilitation differ across sub-groups of participants [38]. In our trial we will determine if treatment effects are influenced by fracture severity and by the age and gender of participants.

Several strategies will be implemented in order to ensure data quality. Assessors and treating physiotherapists will be adequately trained before they work on the trial. Compliance with the trial protocol will be closely monitored by an on-site associate investigator. Data forms and processing will be regularly scrutinised for accuracy and completeness. All data entry will be double-checked for accuracy. After the trial, the security of randomisation will be evaluated by comparing actual group allocations with a record of the randomisation schedule.

Recruitment commenced in December 2010 and trial completion (follow-up of all participants) is planned for April 2013. The results are expected to be available by the end of 2013.

## Competing interests

The authors declare that they have no competing interests.

## Authors' contributions

AM, CL, RH and MH are the principal investigators - together they conceived and designed the trial and procured funding. PB drafted the first version of the manuscript. All authors contributed to the writing of the manuscript. All authors read and approved the final version of the manuscript.

The National Health and Medical Research Council (NHMRC), Australia, provides funding for this trial. CL and RH are funded by fellowships from the NHMRC.

## Pre-publication history

The pre-publication history for this paper can be accessed here:

http://www.biomedcentral.com/1471-2474/12/148/prepub
